# Design and Implementation of a Multinational Computerized Patient Survey: The European Paediatric Orthopaedic Society Down Syndrome Survey

**DOI:** 10.1100/tsw.2004.26

**Published:** 2004-04-20

**Authors:** David M. Steinberg, Moshe Kaplan, Joav Merrick, Shlomo Wientroub

**Affiliations:** ^1^ Department of Statistics and Operations Research, Tel Aviv University, Israel; ^2^ School of Computer Science, Tel Aviv University, Israel; ^3^ National Institute of Child Health and Human Development, Office of the Medical Director, Division for Mental Retardation, Ministry of Social Affairs, Jerusalem and Zusman Child Development Center and Divisions of Pediatrics and Community Health, Ben Gurion University, Beer-Sheva, Israel; ^4^ Department of Pediatric Orthopaedics, Dana Children's Hospital, Sourasky Medical Center, Sackler School of Medicine, Tel Aviv University, Israel

**Keywords:** multinational computerized patient survey, musculoskeletal problems, Down syndrome, pediatric orthopedics, human development, public health, Israel

## Abstract

In 1997, the European Paediatric Orthopaedic Society (EPOS) initiated an international survey of musculoskeletal problems in individuals with Down syndrome. The goals of the study were far-reaching and ambitious in order to obtain a clear picture of the variety of orthopedic problems associated with Down syndrome, to relate their occurrence to other medical conditions, and to record and compare methods of treatment. The survey was to be international in scope, including orthopedic surgeons and departments across Europe and outside Europe. The study was coordinated from the Tel Aviv University in Israel as a multicenter study using electronic technology. This paper describes the organization and implementation of the EPOS Down Syndrome Survey, with special emphasis on the sophisticated electronic data entry instrument that was developed especially for the survey. It is concluded that computerized data entry forms like the one that we developed for the EPOS Down Syndrome Survey are valuable tools for medical surveys. They are especially important for multicenter and multinational studies.

## INTRODUCTION

In 1997, the European Paediatric Orthopaedic Society (EPOS) initiated an international survey of musculoskeletal problems in individuals with Down syndrome. The goals of the study were ambitious: to obtain a widespread picture of the variety of orthopedic problems associated with Down syndrome, to relate their occurrence to other medical conditions, and to record and compare methods of treatment. The survey was to be international in scope, including orthopedic surgeons and departments across Europe and outside Europe.

Our purpose in this short communication is to describe the organization and implementation of the EPOS Down Syndrome Survey, with special emphasis on the sophisticated electronic data entry instrument that was developed especially for the survey.

## STUDY POPULATION

The study planners desired to obtain as broad a picture as possible of orthopedic problems among those with Down syndrome. Thus all individuals with Down syndrome were eligible for entry into the study. In many of the participating countries, a member of EPOS took responsibility for coordinating the national data collection efforts. The study did not have access to national registries of individuals with Down syndrome, which precluded the option of probability sampling. Thus it was expected that the study would be biased toward individuals with orthopedic conditions. Nonetheless, this sample provides valuable information as to treatment modalities and results. A sampling frame was implemented in Israel in an attempt to add information about the prevalence of conditions.

That sample included a near census of individuals above 21 years of age living in residential care centers and a sample drawn from the lists of the Israel Down Syndrome Association (YATED) family support organization, which includes a large percentage of cases in the Jewish population in Israel[1].

## SURVEY INSTRUMENT

The data collection goals were extensive. The primary focus was on the subject’s musculoskeletal conditions. Data were also assembled on general health status, parents’ and siblings’ health status, pregnancy and birth, molecular genetics, and systemic problems.

A special questionnaire was developed for the survey. The questionnaire had a main form filled out for each person with extensive medical history data. The main form included sections on musculoskeletal history and management, fractures, status at follow-up, pain, laxity profile, posture and tonus, gait and balance, fixed contractures, treatment procedures and results, associated medical problems, pregancy and birth, living conditions, communication, and sports activity. The main form also included information on musculoskeletal problems and general health status of parents and siblings. The musculoskeletal report was grouped into several major sections: cervical spine, lower spine, hip, knee, hand, and foot. Six special forms were provided for detailed information on particular conditions (atlanto-axial instability, lower cervical spine, scoliosis, hip, patello-femoral instability, and club foot/vertical talus). Fig. 1 shows an overview of the data collection, with groupings for family history and subject data, including general information and special forms when relevant.

### The Electronic Form

Computerized data collection forms were developed using the Microsoft™ Access database software. The forms provided a user-friendly interface that guided the data entry process. Fig. 2 shows the initial data collection screen. Blank spaces were provided to enter basic information like the patient’s name, hospital id number, etc. “Multiple choice” items like sex could be entered with a click of the mouse. For some of these items, the physician was asked to “check” the correct box. For other items, he could open a “pop-up menu” by clicking on an arrow and then click on the mouse to choose the correct entry from the menu. Some of these menus included the option of entering an additional item beyond those in the list.

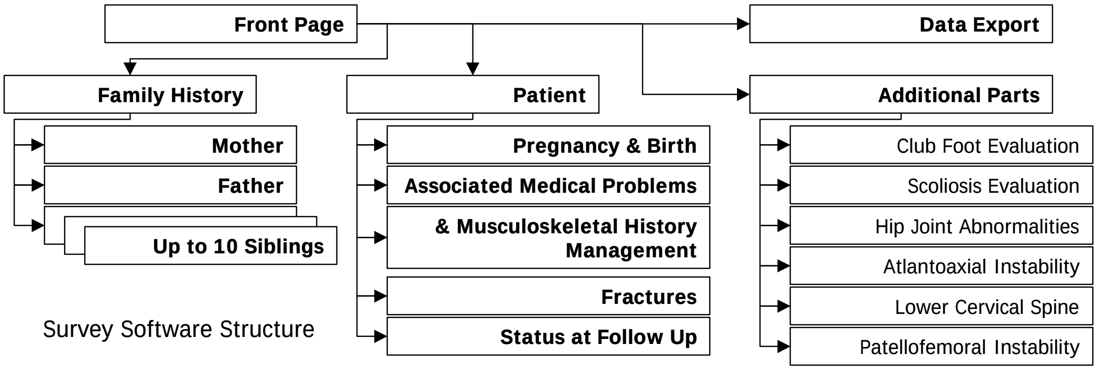

**FIGURE 1.** An overview of the information collected on each subject.

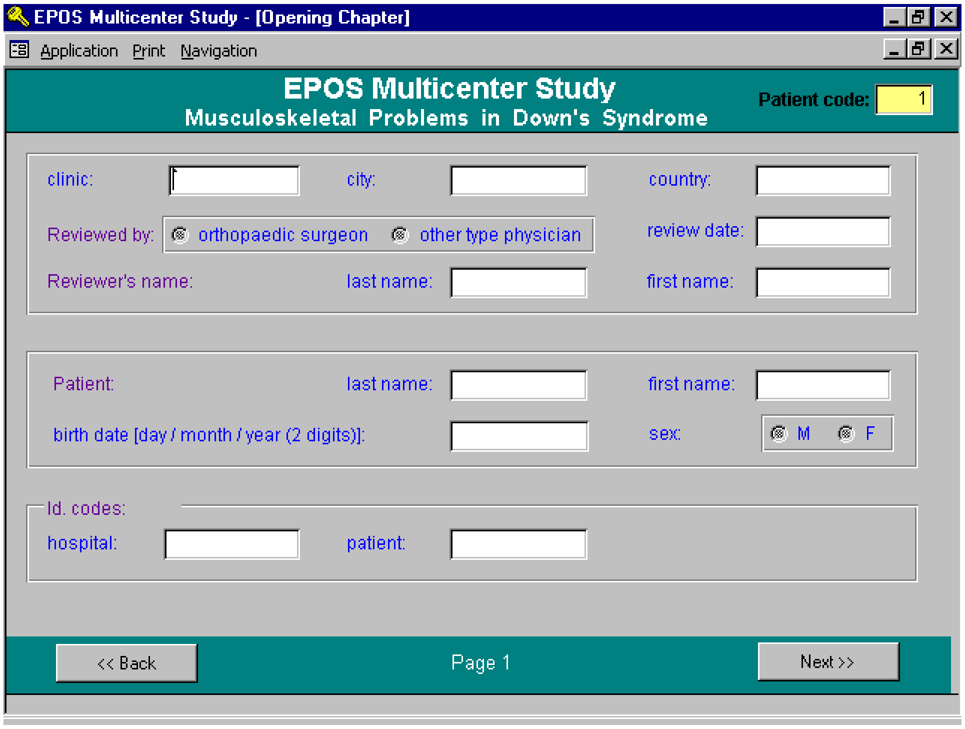

**FIGURE 2**. The initial data entry screen in the electronic form.

The initial data entry screen for musculoskeletal history and management is shown in Fig. 3. This part of the form began with spinal problems. Subsequent screens collected data for other organs.

The complete computerized form was divided into subsections along the same lines specified in the section on medical data. The data for each patient were typically entered sequentially, as in filling out a paper form from beginning to end. Completing a multiple choice item, or hitting the "Enter" key after completing a free text item, automatically moved the cursor to the next item and brought up the next screen if the current one was completed. Each screen included a menu for moving back to the previous page or to the next page. The form also included a "navigation" menu (in the second line from the top of the screen, see Fig. 4) that enabled the physician to move directly from the initial screen to any of the subsections via two mouse clicks. The navigation feature was especially useful when the data entry was interrupted midpatient or when additional information on a patient was added after the initial data entry had been completed.

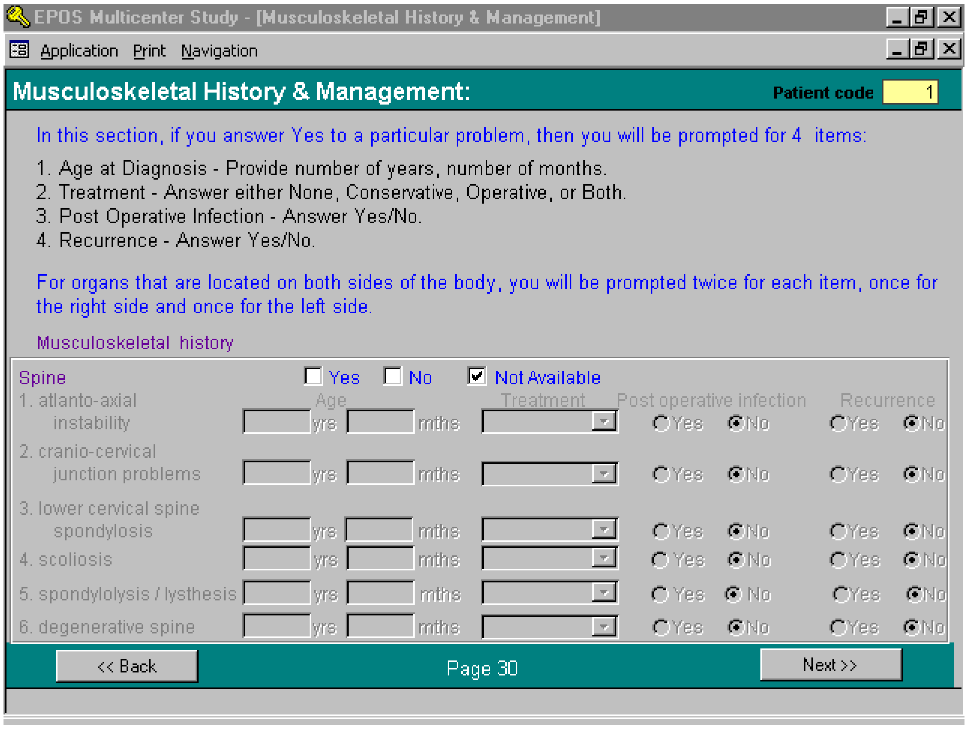

**FIGURE 3**. The initial data entry screen for musculoskeletal history and management.

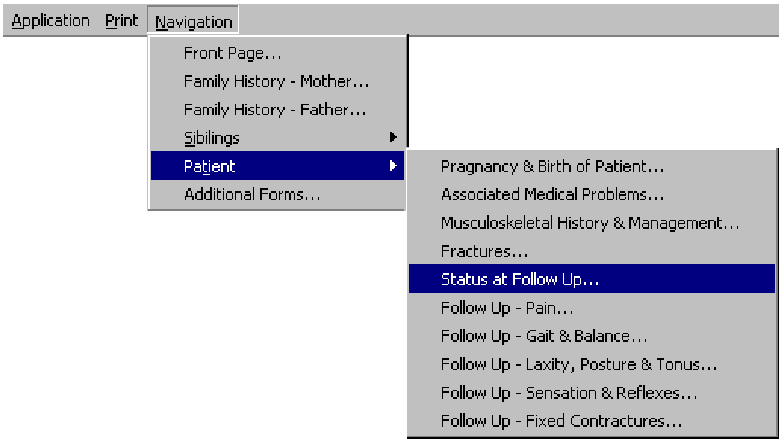

**FIGURE 4.** Navigation menus.

## Required Items

Some data items were required. If a required item was left blank, attempts to progress to the next item by hitting the “Enter” key or to the next screen resulted in a warning to the user that the current item must first be completed. For example, the orthopedic screens typically began with a general question on the existence of medical problems. In Fig. 3, for example, the user was first prompted to note whether or not the subject has any spinal problems. If the answer to that item was affirmative, the user was required to specify at least one of the particular problems listed on the screen (the specific conditions listed as numbers 1 through 6 in Fig. 3).

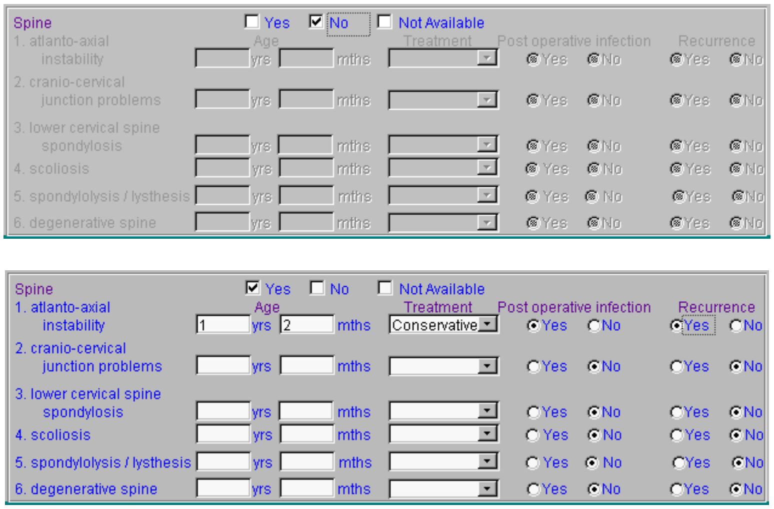

**FIGURE 5.** Implementation of conditional items in the form for spinal problems. In the form shown on the top, the box for no problems has been checked and the detailed questions on spinal problems are dimmed. Checking the Yes box, as in the form on the bottom, lights up the questions, prompting the physician for data.

## Conditional Items

A special feature of our computerized forms was the use of many conditional items, which would be presented to the physician only if a prior item received a positive answer. For example, in the form for spinal problems in Fig. 3, the initial check box was “lit up” but the following specific items were “dimmed”. If the physician checked the “no” box for presence of problems, the specific items were left dimmed and the physician was automatically directed to the next screen, thus skipping over the irrelevant questions. If the physician checked the “yes” box, the remaining items were lit up and the physician was prompted to answer them. Fig. 5 contrasts the page for spinal problems in the dimmed and activated modes.

The special data forms were each designed to be conditional on answers to items in the main data form. Thus, for example, if the physician did not record any hip problems for the patient on the main “hip” screen, the special hip form would be inaccessible for that patient.

The use of conditional items improved data reliability by helping to guarantee internal consistency. It also accelerated data entry by helping physicians to skip rapidly over many items that were not relevant for the particular patient.

## Logic Checks

Many logic checks were also built into the computerized form to improve data reliability. For example, a user who entered 150 cm as the height at birth was presented an error message that the value is not plausible and should be corrected. The software also compared the diagnosis dates for a patient’s medical conditions to the patient’s birth date. If the diagnosis date preceded the birth date the program presented an error message indicating the incorrect chronology of the dates. The automatic logic checks reinforced data integrity by filtering out many errors at the data entry phase. Such checks were especially important for a multinational study such as the EPOS Down Syndrome Survey, where the physical separation between the coordinating center and the participating hospitals and clinics sharply limited the possibility of subsequent review of erroneous entries.

### The “Not Available” Option

The first item on many of the medical conditions asked whether or not the patient has the condition. In addition to “Yes” and “No” answers, we included a “Not Available” option for cases in which the MD does not have definitive information. This item was essential for many of the subsequent statistical analyses, which excluded subjects for whom no data were available. We made “Not Available” the default option to ensure that subjects lacking data would not inadvertently be treated as if they were known to be free of the condition. See Figs. 3 and 5 for examples of this option.

### Software Manual

A simple written manual was prepared for using the computerized data entry form. The manual included instructions on how to install the data entry forms from a CD, how to enter and store the data, including corrections and updates, and how to prepare a data file for submission to the coordinating center.

## DATA COLLECTION AND COORDINATION

Compact discs (CDs) with the computerized forms were sent to EPOS coordinators in each participating country. These individuals contacted additional pediatric orthopedists for input. Ultimately, 16 different countries provided data for the study: Argentina, Austria, Belgium, Brazil, Croatia, the Czech Republic, France, Germany, Israel, Italy, Japan, the Netherlands, Poland, Spain, Switzerland, and the U.K.

The Statistical Laboratory at Tel Aviv University served as the data coordinating center. Completed data files were sent on diskettes or by electronic mail to the Statistical Laboratory. These files were merged into a unified Access™ database. Country codes were assigned as part of the merge process. The database was used to generate additional files for the statistical analyses.

Fig. 6 shows the overall data flow for the project. The data entry software was distributed to numerous countries (1), data were collected and entered into the software (2), data were sent by diskette or electronic mail to the Statistical Laboratory (3), where they were combined into a unified database (4), and used to generate data sets for statistical analysis (5).

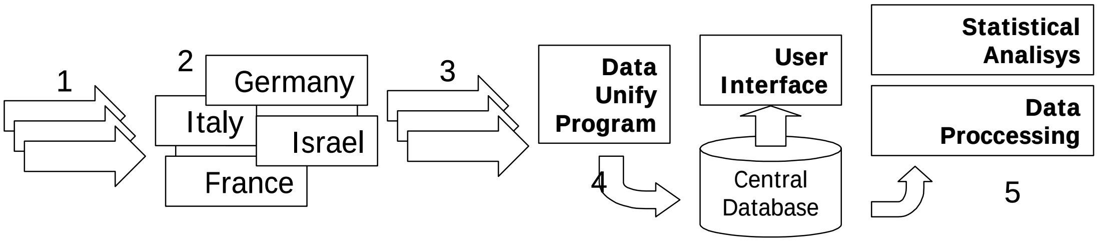

**FIGURE 6**. A schematic flow of the data collection and analysis process.

## CONCLUSIONS

The computerized data entry procedure was extremely effective in the EPOS Down Syndrome Survey. All the medical professionals who used the software to enter data found it convenient and easy to use. The computerized forms were able to minimize many potential data quality problems. Obviously implausible entries were screened out by built-in logic checks. Data were keyed in to the computer at the medical centers where the subjects were treated, eliminating many errors. Had we collected the data on handwritten paper forms and done the computer entry at the data coordinating center, numerous errors would certainly have occurred. Moreover, the difficulty in communicating directly with the participating medical centers would have made it nearly impossible to correct such errors.

The computerized form streamlined the data entry process by automatically directing the user to skip over unnecessary items.

Several important decisions are necessary in developing a computerized survey, including protocols for storing the data and for providing an interface to the data. Data storage may be accomplished using regular files or a database, which includes built-in methods to handle data like deleting, inserting, grouping, and sorting. We chose to use a database and are convinced that the tools were valuable in improving our data handling.

The available tools to create a data interface are HTML pages, which are mostly used on the World Wide Web, and Rapid Application Development environments, which enable both presentation of screens and use of computer code to manage users. Our selection was Microsoft™ Access database software, which includes both a database and an environment to quickly develop forms and code.

A final important decision is whether to use single-station architecture or a server and client system. In the former, each user installs and runs the data collection software on their own personal computer. In a server and client system, the data and software reside on a central computer, which the user must access over the Internet whenever he or she wants to enter data. We preferred the single-station mode due to its flexibility and out of concern for possible data security problems associated with transmitting data over the Internet.

We believe that computerized data entry forms like the one that we developed for the EPOS Down Syndrome Survey are valuable tools for medical surveys. They are especially important for diffuse multicenter, and even multinational studies, like the one we conducted.

## BIOSKETCHES

**David M. Steinberg, PhD**, Professor, Department of Statistics and Operations Research, Tel Aviv University, IL-69978 Tel Aviv, Israel. E-mail: dms@post.tau.ac.il

**Moshe Kaplan, BSc,** is a graduate student of the Engineering faculty at the Tel Aviv University, Israel, and software designer of systems in education and medical fields. Currently director of a software team dealing with large software programs. E-mail: mks@post.tau.ac.il

**Joav Merrick, MD, DMSc,** is Professor of Child Health and Human Development affiliated with the Zusman Child Development Center, Division of Pediatrics and Community Health at the Ben Gurion University, Beer-Sheva, Israel; the Medical Director of the Division for Mental Retardation, Ministry of Social Affairs, Jerusalem; and the Founder and Director of the National Institute of Child Health and Human Development. He has numerous publications in the field of child and human development, rehabilitation, intellectual disability, disability, health, welfare, abuse, advocacy, quality of life, and prevention. Dr. Merrick received the Peter Sabroe Child Award for outstanding work on behalf of Danish Children in 1985 and the International LEGO-Prize ("The Children's Nobel Prize") for an extraordinary contribution towards improvement in child welfare and well being in 1987. E-mail: jmerrick@internet-zahav.net. Website: www.nichd-israel.com

**Shlomo Wientroub, MD,** Professor of Pediatric Surgery and Chairman, Department of Pediatric Orthopaedics, Dana Children's Hospital, Sourasky Medical Center, Sackler School of Medicine, Tel Aviv University, Israel. E-mail: shlomowi@tasmc.health.gov.il
